# Treatment of Gram-negative pneumonia in the critical care setting: is the beta-lactam antibiotic backbone broken beyond repair?

**DOI:** 10.1186/s13054-016-1197-5

**Published:** 2016-01-29

**Authors:** Matteo Bassetti, Tobias Welte, Richard G. Wunderink

**Affiliations:** 1 Santa Maria Misericordia University Hospital, Piazzale S. Maria Misericordia 15, 33100 Udine, Italy; 2 Department of Respiratory Medicine, Hannover Medical School, Carl-Neuberg-Strasse 1, 30625 Hannover, Germany; 3 Northwestern University Feinberg School of Medicine, 676 North St. Clair Street, Arkes 14-015, Chicago, IL 60611 USA

## Abstract

Beta-lactam antibiotics form the backbone of treatment for Gram-negative pneumonia in mechanically ventilated patients in the intensive care unit. However, this beta-lactam antibiotic backbone is increasingly under pressure from emerging resistance across all geographical regions, and health-care professionals in many countries are rapidly running out of effective treatment options. Even in regions that currently have only low levels of resistance, the effects of globalization are likely to increase local pressures on the beta-lactam antibiotic backbone in the near future. Therefore, clinicians are increasingly faced with a difficult balancing act: the need to prescribe adequate and appropriate antibiotic therapy while reducing the emergence of resistance and the overuse of antibiotics. In this review, we explore the burden of Gram-negative pneumonia in the critical care setting and the pressure that antibiotic resistance places on current empiric therapy regimens (and the beta-lactam antibiotic backbone) in this patient population. New treatment approaches, such as systemic and inhaled antibiotic alternatives, are on the horizon and are likely to help tackle the rising levels of beta-lactam antibiotic resistance. In the meantime, it is imperative that the beta-lactam antibiotic backbone of currently available antibiotics be supported through stringent antibiotic stewardship programs.

## The ‘antibiotic backbone’

Antibiotics for the treatment of serious bacterial infections have immeasurable benefits for the critically ill patient and have greatly reduced morbidity and mortality since their widespread adoption in the 1950s. However, global overuse of these drugs has led to the development of resistance and decreased effectiveness, making it increasingly difficult to choose appropriate antibiotic therapy options for life-threatening infections, such as pneumonia, in the intensive care unit (ICU).

The term ‘antibiotic backbone’ was coined to describe those drugs that form the foundation of antimicrobial therapy; these backbone antibiotics treat most bacterial infections effectively, and appropriate empirical use reduces both mortality and the emergence of resistance [1–3]. The choice of antibiotics is based on many factors, including recommendations from guidelines [4–6] and evidence-based reviews [1, 7]. Currently, the antibiotic backbone for the treatment of pneumonia consists of drugs from the beta-lactam class. However, increasing resistance rates are beginning to seriously limit the clinical utility of beta-lactams [8, 9]. Therefore, in this review, we explore the current use of beta-lactam antibiotics, the challenges posed by increasing resistance rates, and how the beta-lactam antibiotic backbone might be strengthened in the future. The article reflects our best knowledge and shared professional opinion of the currently available evidence on the topics discussed, but an exhaustive or systematic review of the literature would be outside of its scope.

## Pneumonia in the intensive care unit

### Burden and mortality

Hospital-acquired pneumonia (HAP) is one of the most common infections in the ICU [10, 11]; published rates ranged from five to more than 20 cases per 1000 hospital admissions [11, 12]. HAP is also associated with high morbidity and mortality as well as a high health-care and economic burden [13]. Up to 44 % of all HAP infections are acquired in the ICU; of these, up to 90 % are ventilator-associated pneumonia (VAP) [12, 14]. VAP is currently defined as pneumonia arising more than 48–72 hours after endotracheal intubation [4]. However, diagnosis of HAP/VAP may be subjective and open to interpretation. The resulting considerable variation in reporting makes comparison of data between institutions difficult [15]. Indeed, the magnitude in variability shows that current surveillance definitions of VAP perform poorly in the clinical setting and suggests that a new, more objective definition is required [15]. Regardless of criteria for diagnosis, VAP is associated with poor clinical outcomes, including increased morbidity and mortality [12, 16], increased hospital stay, and increased duration of mechanical ventilation [17]; notably, the duration of ventilation is an important risk factor in the development of VAP [18].

In addition to recognizing HAP and VAP, current guidelines recognize health care-associated pneumonia (HCAP); this classification includes patients with pneumonia who were recently hospitalized in an acute care hospital, resided in a nursing home or long-term care facility, or received recent intravenous (IV) antibiotic therapy [4]. Many of these patients have bacterial etiologies that require the same broad-spectrum beta-lactam-based treatment as HAP/VAP.

Mortality rates for HAP/VAP are influenced by many factors, such as patient age, presence of underlying comorbidities, adequacy of antibiotic treatment given, and, for those with VAP, time on mechanical ventilation [17]. Mortality rates vary from study to study, but up to one third of all HAP-related deaths are directly attributable to pneumonia [19]. Crude mortality rates for VAP range from 24 % to 50 % but can reach up to 76 % in specific settings with high-risk pathogens [20]. Attributable mortality rates for VAP are difficult to determine but may be less than 10 % [12, 16, 21], given that VAP occurs in patients who have already suffered a critical illness.

### Etiology

**Fig. 1:**
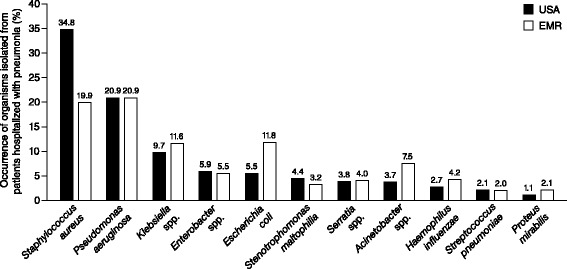
Etiology of pneumonia in the hospital and critical care setting. *EMR* Europe and Mediterranean Region. Data from Sader et al. [23] (2014)

The distribution of pathogens that comprise the etiology of pneumonia (including HAP, VAP, and HCAP) varies from region to region [22, 23]. The 2009–2012 SENTRY antimicrobial surveillance program included data from nearly 13,000 isolates from patients hospitalized with pneumonia, collected from 53 hospitals across the USA and Europe. The same top 11 organisms were observed in both geographic regions, albeit in different rank orders (Fig. 1); the most frequently observed Gram-positive pathogen was *Staphylococcus aureus*, whereas the most common Gram-negative pathogen was *Pseudomonas aeruginosa*. Other common Gram-negative pathogens included *Klebsiella* spp., *Enterobacter* spp., *Acinetobacter* spp., and *Escherichia coli* [23].

## The antibiotic backbone under strain and the rise of resistance

**Fig. 2:**
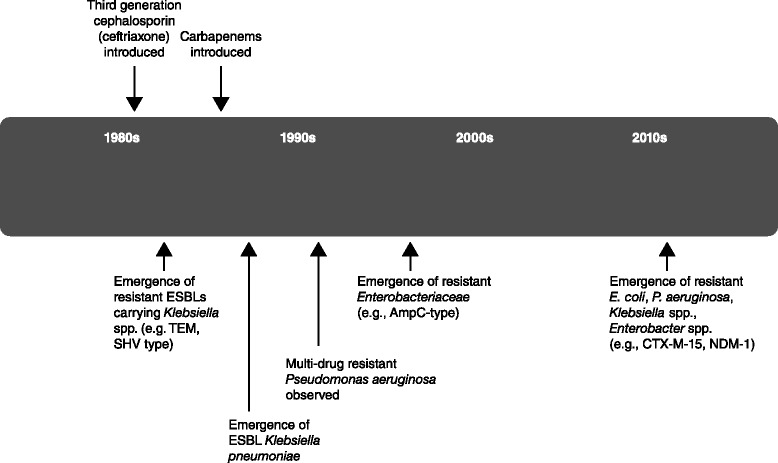
Evolving resistance patterns for Gram-negative pathogens associated with pneumonia in the critical care patient. Approximate years in which resistant organisms were identified are shown. *AmpC* AmpC-producing *Enterobacteriaceae*, *ESBL* extended-spectrum β-lactamase-producing *Enterobacteriaceae*, *NDM-1* New Delhi metallo-β-lactamase-1-producing *Enterobacteriaceae*. Adapted from [33] and [94]

To treat pneumonia in the ICU, current guideline recommendations focus on the initiation of effective and timely empiric antibiotic therapy combined with resuscitation and supportive measures. Beta-lactam antibiotics alone or as part of a combination regimen are the mainstay of empirical antibiotic guideline recommendations [4–6]. Despite the availability of beta-lactam treatment options, clinical cure rates for HAP/VAP rarely exceed 60 %, and recurrence rates are high [24–26]. The beta-lactam antibiotic backbone is under strain from the increased prevalence and variety of bacterial antibiotic resistance. The frequency of multi-drug-resistant (MDR) pathogens has accelerated dramatically in recent years (Fig. 2) [27–29]. The increasing frequency and variety of resistance patterns have also necessitated the introduction of definitions for ‘extensively drug-resistant’ pathogens (non-susceptibility to at least one agent in all but two or fewer antimicrobial categories) and ‘pandrug-resistant’ pathogens (non-susceptibility to all agents in all antimicrobial categories) [30].

Rates and patterns of antibiotic resistance differ between countries, regions, and hospitals [22, 23, 31], and high levels of resistance have been observed across all main strains of pathogens. For example, the SENTRY study reported reduced susceptibility of *P. aeruginosa* to most antimicrobials tested, including ceftazidime (79.6/68.7 % susceptibility in USA/Europe), meropenem (76.3/65.8 %), and piperacillin/tazobactam (72.9/63.9 %). Furthermore, *Klebsiella* spp. showed extended-spectrum beta-lactamase (ESBL) phenotype prevalences of 19.5 % and 35.1 % in the USA and Europe, respectively; meropenem was active against 62.3 % and 78.7 % of ESBL *Klebsiella* spp. [23]. These concerning levels of beta-lactam resistance in Gram-negative pathogens are reflected in a recent comprehensive report issued by the World Health Organization (WHO), which included data from 129 WHO member states on nine bacteria–antibacterial drug combinations [22]; the report included global resistance data on a number of Gram-positive and Gram-negative pathogens that are commonly involved in pneumonia (Table 1). WHO member states reported high rates of beta-lactam resistance, including resistance to third-generation cephalosporins and carbapenems in *K. pneumoniae* [22]. In addition to these resistance mechanisms, new mechanisms—e.g., New Delhi metallo-beta-lactamases (NDM-1 and NDM-4)—are emerging [32]. Given the global increase in antibiotic resistance rates and the striking variation between regions, it is clearly important that treatment guidelines be adopted on the basis of local surveillance and epidemiology data, be validated, and be applied with consideration for new antimicrobial drugs [4–6].

**Table 1:** World Health Organization-reported beta-lactam resistance rates (percentage) in common bacterial pathogens that can cause pneumonia (most recent data as reported 2013)

Pathogen	Africa	Americas	Eastern Mediterranean	Europe	South East Asia	Western Pacific	WHO key points
*Staphylococcus aureus* (beta-lactam resistant; i.e., MRSA)	12–80	21–90	10–53	0.3–60	10–26	4–84	Data on MRSA proportions among *S. aureus* were obtained from 44 % of member states; most reported MRSA proportions exceed 20 % in all WHO regions and even exceed 80 % in some reports.
*Streptococcus pneumoniae* (penicillin-resistant)	3–16	0–48	13–34	0–61	47–48	17–64	Data were obtained from only 35 % of member states; non-susceptibility to penicillin has been detected in all WHO regions.
*Escherichia coli* (resistant to third-generation cephalosporins)	2–70	0–48	22–63	3–82	16–68	0–77	Data on *E. coli* resistance to third-generation cephalosporins were obtained from 44 % of member states; reports consistently disclosed high resistance rates to the last-generation drugs commonly used to treat serious infections.
*Klebsiella pneumoniae* (resistant to third-generation cephalosporins)	8–77	4–71	22–50	2–82	34–81	1–72	The majority of sources reported more than 30 % resistance in *K. pneumoniae* to third-generation cephalosporins in the sampled populations; resistance proportions exceeding 50 % were reported from all WHO regions.
*Klebsiella pneumoniae* (resistant to carbapenems)	0–4	0–11	0–54	0–68	0–8	0–8	Rates of carbapenem resistance exceeding 50 % have been reported in some patient groups, for which few, if any, alternative treatment options are available.

Resistance ranges are based on national data reported to the WHO [22]

*MRSA* methicillin-resistant *S. aureus*, *WHO* World Health Organization

## The consequences of a broken beta-lactam antibiotic backbone

A broken beta-lactam antibiotic backbone means that bacterial infections cannot be reliably treated empirically with a particular antibiotic regimen. The emergence of resistance to beta-lactam antibiotics has exerted significant pressure on the antibiotic backbone, resulting in increased morbidity, mortality, and health-care costs [28–31, 33, 34]. Indeed, ineffective therapy can lead to many complications, including adverse events, superinfections [35], emergence of resistance [36, 37], increased ICU or hospital length of stay [37, 38], increased hospitalization costs [38], antibiotic-induced organ dysfunction [39], and often the need to prescribe additional antibiotics. Patients with MDR Gram-negative pneumonia may be at greater risk of death than those infected with a non-MDR organism. For example, in a recent international multicenter study in patients with *P. aeruginosa* nosocomial pneumonia, infection with MDR strains was associated with significantly higher in-hospital mortality compared with infection with non-MDR strains (44.7 % versus 31.7 %, *P* < 0.001) [40]. However, the overall evidence on a potential association of mortality outcomes with MDR infection in patients with pneumonia is still unclear [41, 42].

## Current strategies to brace the antibiotic backbone

How can the pressure on the beta-lactam antibiotic backbone be relieved? Current protective strategies include antimicrobial stewardship, the optimization of beta-lactam pharmacokinetic/pharmacodynamic (PK/PD) exposure, and the use of combination therapy with other drug classes (e.g., concurrent administration of systemic aminoglycosides).

### Antimicrobial stewardship

Ideally, antibiotic stewardship in ICUs should include the rapid identification of bacterial etiology, optimization of treatment based on PK/PD characteristics of the antibiotic(s), avoidance of unnecessarily broad-spectrum antibiotics, shortening of treatment duration, and reduction in the number of patients receiving antibiotic therapy for non-infectious syndromes [43, 44]. To achieve this, ICUs should continuously collect data and adjust prescribing of beta-lactam backbone antibiotics according to their local resistance patterns [45]. Delayed appropriate therapy is consistently associated with worse outcomes [8, 46–48]. In contrast, early and appropriate empiric broad-spectrum therapy, followed by de-escalation, is generally associated with improved clinical outcomes for pneumonia [4, 49, 50]. To achieve adequate therapy, it is necessary to select the correct antibiotic(s), an optimal dose, and the correct route of administration (oral, IV, or aerosol) to ensure antibiotic penetration to the site of infection [4].

Guidelines advise that de-escalation of antibiotics be considered once information is available on the results of lower respiratory tract cultures and the patient’s clinical response [4]. A meta-analysis that included three studies in patients with VAP showed that, in some patients, the use of short-course (2- to 3-day) therapy as a de-escalation approach (compared with prolonged-course therapy) was not associated with an increase in mortality, duration of mechanical ventilation, or hospital stay, particularly in those not infected with non-fermenting Gram-negative bacilli [51]. While the optimal length of antibiotic therapy for critically ill patients with VAP due to non-fermenting Gram-negative bacilli remains an unsolved issue [52], clinicians should strive to stop therapy as soon as possible.

De-escalation of therapy may be guided by scores such as the clinical pulmonary infection score (CPIS) [53]. In a randomized controlled trial (RCT), 81 patients with a CPIS of less than 6 (implying low likelihood of pneumonia) were randomly assigned to receive either standard therapy (at discretion of physician) or ciprofloxacin monotherapy, with re-evaluation at day 3; ciprofloxacin was discontinued if CPIS remained less than 6 at day 3. Mortality, length of ICU stay, and development of resistance did not differ significantly between treatments, actually favoring the ciprofloxacin monotherapy arm.

Biomarkers offer an alternative method to shorten duration of therapy. Several large randomized trials in critically ill patients of antibiotic duration based on falling procalcitonin levels have demonstrated that this approach can safely shorten duration without adverse consequences [54]. HAP/VAP were common types of infection in these studies.

### Optimizing beta-lactam pharmacokinetic/pharmacodynamic exposure

The beta-lactam antibiotic backbone may be further supported by optimizing beta-lactam antibiotic PK/PD exposure, which could include considerations of the length of infusion and the use of appropriate loading doses. The pharmacokinetics of systemic antibiotics in critically ill patients are highly variable because of patients’ physiological changes affecting drug absorption, distribution, metabolism, and elimination [55]. These changes make the correct dosing of antibiotics in these patients very challenging and can result in the delivery of sub-therapeutic or toxic drug concentrations [56, 57]. The DALI (Defining Antibiotic Levels in Intensive Care Patients) multinational ICU study was a pharmacokinetic point-prevalence study including eight systemically administered beta-lactam antibiotics [58]. Overall, 16 % of patients in the study did not achieve free antibiotic concentrations sufficiently greater than the minimum inhibitory concentration (MIC) required to ensure a positive clinical outcome. Patients who had concentrations above the MIC for at least 50 % or 100 % of the dosing interval were more likely to have a positive clinical outcome (odds ratio 1.02 and 1.56, respectively; *P* < 0.03) [58]. In line with this, a recent study by MacVane et al. demonstrated that, in patients with VAP caused by Gram-negative bacilli, a serum exposure greater than 53 % *f*T > MIC was significantly associated with a favorable microbiological response (eradication or presumed eradication of pathogen) to anti-pseudomonal cephalosporins [59]. Similarly, a study by Muller et al. found that ceftobiprole exposure (*f*T > MIC of greater than 62 % of the dosing interval) strongly correlated with microbiological eradication and clinical cure in patients with nosocomial pneumonia [60]. These and other studies (e.g., Crandon et al. [61]) emphasize the importance of considering exposure–response profiles when optimizing drug therapy in these patient groups [59–61].

One way to optimize beta-lactam antibiotic dosing may be the use of prolonged or continuous infusion, which could benefit critically ill patients with severe illness [62–64]. Continuous infusion of beta-lactams often also includes the use of a loading dose to ensure early attainment of target concentrations exceeding the MIC [63]. Currently, clinical evidence is still unclear as to whether it is better to give beta-lactam antibiotics by traditional intermittent bolus dosing or continuous infusion. Theoretically, continuous infusion of beta-lactam antibiotics should be advantageous, because it produces more sustained antibiotic concentrations above the MIC (a key measure to describe the bacterial kill characteristics of beta-lactams) [62]. A number of reports suggest that continuous infusion has clinical benefits (reviewed in [62–64]). For example, one RCT in 30 patients with severe sepsis reported that continuous administration of beta-lactam antibiotics achieved higher plasma antibiotic concentrations than intermittent administration, with associated improvements in clinical cure (70 % versus 43 %, *P* = 0.037) [65]; however, a more recent and larger (n = 432) trial by the same research group, again conducted in critically ill patients with severe sepsis, reported no difference in outcomes between beta-lactam antibiotic administration by continuous and intermittent infusion [66]. Indeed, two comprehensive systematic meta-analyses suggest that, overall, clinical studies in critically ill patients with acute infections have not yet conclusively demonstrated the benefits of continuous over bolus infusion [63, 64]; one of these analyses did suggest a benefit in mortality outcomes [64], whereas the other did not [63]. Clearly, further evidence, particularly from large RCTs, is needed [62–64].

Another potential strategy to help overcome the antibiotic dosing challenges in patients in the ICU may be therapeutic drug monitoring (TDM). However, a recent systematic review found that only a small number of the included studies reported that TDM may improve beta-lactam dosing in critically ill patients [67]. The systematic review also found little agreement between studies on the pharmacodynamic targets for optimizing antibiotic therapy. Clearly, more data are needed before any potential clinical benefits of TDM can be established [67, 68]. However, even if clinical improvements can be conclusively demonstrated, TDM is currently not routinely available in most clinical laboratories.

### Other strategies

Evidence suggests that the pressure on beta-lactams could be relieved by support from other antibiotic classes (for example, using combination therapy). A systematic review comparing outcomes of combination therapy and monotherapy for carbapenem-resistant *Enterobacteriaceae* (CRE)-causing respiratory infections found that combination therapy was associated with significantly lower clinical failure rates (29 % versus 67 %, *P* = 0.03) [69].

In contrast, in a study that included 740 mechanically ventilated patients with suspected VAP, monotherapy was associated with outcomes similar to those of combination therapy [70]. Unfortunately, most high-risk patients were systematically excluded from this study. However, in the subgroup with MDR pathogens, combination therapy demonstrated a trend toward improved outcomes. In another study that evaluated monotherapy versus combination therapy in patients with VAP at low risk for difficult-to-treat pathogens [71], infection could be effectively managed with antibiotic monotherapy; outcomes of ICU stay, clinical response, and emergence of resistance were similar to those seen with combination therapy [71]. However, in the subgroup of patients with VAP due to *Pseudomonas* species, *Acinetobacter* species, or MDR Gram-negative bacilli (n = 56), combination therapy demonstrated better microbiological outcomes and was associated with shorter durations of mechanical ventilation and ICU stay, although it should be noted that the study was not powered to demonstrate statistical significance for this comparison [71]. Finally, a Cochrane systematic review compared beta-lactam monotherapy with beta-lactam–aminoglycoside IV combination therapy in patients with sepsis (including patients with pneumonia) but found no change in all-cause mortality or clinical failure between mono- and combination therapy [72]. The review also found that combination therapy with an IV beta-lactam and an IV aminoglycoside carried a significant risk of nephrotoxicity (combined risk ratio in favor of monotherapy: 0.30, 95 % confidence interval 0.23 to 0.39) [72].

## Future strategies to brace the antibiotic backbone

Biomarkers are under investigation as a tool to reduce unnecessary antibiotic prescribing and further facilitate antimicrobial stewardship [73–75]. In one recent study, monitoring of procalcitonin levels (obtained upon ICU admission or after a new suspected infection, followed by a second level measurement 48 hours later) was associated with achieving significantly lower antibiotic exposure (10 versus 13 days, *P* < 0.003) compared with using no monitoring [76]; procalcitonin monitoring was also associated with significant reductions in length of hospital stay, hospital readmission, and relapse of infection [76]. These findings are supported by several large randomized trials in critically ill patients [54].

New tools for the rapid detection of antibiotic resistance may soon aid physicians in predicting the effectiveness of empiric antibiotic therapy and enable them to quickly readjust the treatment regimen [77]. Balancing the need to confirm the pathogen(s) responsible for infection with the need to initiate prompt empiric therapy has made it difficult to select an appropriate beta-lactam antibiotic without promoting resistance. The rapid multiplex polymerase chain reaction-based Unyvero pneumonia application (UPA) assay was evaluated in 49 patients with mild–severe nosocomial pneumonia; multiple pathogens were detected in 55 % of patients tested but were detected in only 8 % of patients following traditional culture techniques [78]. In addition, UPA detects 13 different resistance genes, potentially permitting initial empiric treatment to be tailored within 6 hours. Although such technology is promising, further research is required to confirm that the detected organisms represent true pathogens rather than colonizing bacteria. Also, not all of the multiple resistance mechanisms in Gram-negative bacteria may be detected with polymerase chain reaction-based diagnostics. In addition, the presence of a specific resistance gene has not been conclusively demonstrated to correlate with clinical response to the associated antibiotic. This is particularly true for the various beta-lactamases that are very substrate-specific (i.e., those that mainly affect only certain beta-lactams but not others).

Another approach for optimizing antibiotic concentrations involves the delivery of high drug concentrations to the lung via aerosolization [79, 80]. The advantages of targeted delivery include drug concentrations in the lung that greatly exceed the MIC, a strategy that could reduce the emergence of resistance, coupled with low serum concentrations that minimize systemic toxicity [79–81]. Until recently, devices used to deliver aerosols were limited by relatively poor delivery efficiency [82] and lack of an aerosol-optimized formulation (i.e., an IV formulation was given as inhalation) [81]. These limitations both impede drug delivery and contribute to adverse effects such as cough or bronchoconstriction. The development of high-efficiency nebulizers (e.g., vibrating mesh technology or adaptive nebulizers) combined with optimized formulations with particles of a size best suited to increased deposition in the lungs (1–5 μm) [79, 83] have the potential to enhance delivery efficiency and to overcome the limitations of previous aerosolized antibiotic treatments [84, 85].

Aerosolized antibiotic therapy is already administered widely in European ICUs during mechanical ventilation [86], despite limited published studies involving critically ill patients with pneumonia. A double-blind, randomized, single-center study of 24 patients at high risk of MDR organisms found that aerosolized gentamicin or amikacin every 8 hours for 2 weeks eradicated a significantly larger proportion of MDR organisms present at baseline compared with placebo (*P* < 0.0001) [87]. Furthermore, patients receiving aerosolized antibiotics had a significantly better clinical response (reduced CPIS and secretion volume) and significantly lower emergence of resistance [87]. A recent meta-analysis also supports aerosolized colistin as an adjunctive therapy to improve clinical, bacteriologic, and infection-related mortality outcomes in patients with VAP compared with IV colistin alone [88]. However, in a study in patients with VAP caused by *P. aeruginosa*, outcomes for aerosolized amikacin + ceftazidime were not significantly improved compared with IV administration of amikacin + ceftazidime (cure, 70 % versus 55 %, *P* = 0.33) [89].

Ongoing, prospective, randomized clinical studies with aerosolized antibiotics appear to be promising. Several options are in development: a combination amikacin–fosfomycin solution delivered via a PARI eFlow inline system, which is currently in phase 2 (ClinicalTrials.gov identifier: NCT01969799); a trial with nebulized tobramycin (ClinicalTrials.gov identifier: NCT01570192), which will explore the use of nebulized tobramycin in combination with IV meropenem and an aminoglycoside (either amikacin or tobramycin); and Amikacin Inhale, an integrated drug–device combination for the delivery of specially formulated Amikacin Inhalation Solution through a Pulmonary Drug Delivery System, which is currently being investigated in two phase III studies (Clinicaltrials.gov identifiers: NCT01799993 and NCT00805168) in combination with standard of care (SOC) treatment to demonstrate clinical superiority versus SOC IV antibiotics (plus aerosol placebo) in Gram-negative pneumonia in intubated and mechanically ventilated patients in the ICU.

A phase 1 trial (n = 9) of combination amikacin–fosfomycin solution delivered via a PARI eFlow inline system reported that both drugs achieved high tracheal aspirate concentrations [90]. Furthermore, no adverse effects in the respiratory tract were observed. A phase 2 trial with this combination antibiotic solution using a vibrating plate nebulizer for 10 days is under way in mechanically ventilated patients with Gram-negative bacterial pneumonia.

In the completed phase 2 studies of Amikacin Inhale [91, 92], microbiologically relevant amikacin concentrations in epithelial lining fluid and tracheal aspirates were achieved after dosing of Amikacin Inhale every 12 hours. Overall, Amikacin Inhale provided high aminoglycoside concentrations in the lung (25-fold higher than reference MIC values for the Gram-negative organisms primarily responsible for pneumonia) while maintaining low serum amikacin concentrations. Data from phase 3 studies are awaited to confirm whether Amikacin Inhale in combination with SOC will improve clinical outcomes over standard IV therapies.

## Conclusions

For physicians treating pneumonia in critically ill patients, the use of the beta-lactam antibiotic backbone is increasingly fraught with uncertainty. Although beta-lactam antibiotics have played an important role in the treatment of pneumonia in the ICU, resistance rates are high; in part, this has been exacerbated by widespread inappropriate prescribing of extended-spectrum beta-lactam antibiotics. The current state of resistance across many antibiotic classes is highly concerning, and the increasing difficulty of treating infections has been described as a ‘major blooming public health crisis’ [93].

The diligent application of antimicrobial stewardship principles, together with optimized PK/PD dosing strategies, can help preserve efficacy of the beta-lactam antibiotics—at least for the time being. However, to relieve the pressure on the beta-lactam antibiotic backbone in the long term, new approaches are urgently needed; these are likely to include the increased use of biomarkers, rapid diagnostic techniques, and new treatment approaches such as inhaled antibiotics.

## Abbreviations

CPIS: Clinical pulmonary infection score
ESBL: Extended-spectrum beta-lactamase
HAP: Hospital-acquired pneumonia
HCAP: Health care-associated pneumonia
ICU: Intensive care unit
IV: Intravenous
MDR: Multi-drug-resistant
MIC: Minimum inhibitory concentration
PK/PD: Pharmacokinetic/pharmacodynamic
RCT: Randomized controlled trial
SOC: Standard of care
TDM: Therapeutic drug monitoring
UPA: Unyvero pneumonia application
VAP: Ventilator-associated pneumonia
WHO: World Health Organization
